# IMRT QA result prediction via MLC transmission decomposition

**DOI:** 10.1002/acm2.13990

**Published:** 2023-04-08

**Authors:** John T. Stasko, William S. Ferris, David P. Adam, Wesley S. Culberson, Sean P. Frigo

**Affiliations:** ^1^ Department of Medical Physics School of Medicine and Public Health University of Wisconsin‐Madison Madison Wisconsin USA; ^2^ Department of Human Oncology School of Medicine and Public Health University of Wisconsin‐Madison Madison Wisconsin USA

**Keywords:** measurement, multi leaf collimator, quality assurance, transmission

## Abstract

**Background:**

Quality assurance measurement of IMRT/VMAT treatment plans is resource intensive, and other more efficient methods to achieve the same confidence are desirable.

**Purpose:**

We aimed to analyze treatment plans in the context of the treatment planning systems that created them, in order to predict which ones will fail a standard quality assurance measurement. To do so, we sought to create a tool external to the treatment planning system that could analyze a set of MLC positions and provide information that could be used to calculate various evaluation metrics.

**Methods:**

The tool was created in Python to read in DICOM plan files and determine the beam fluence fraction incident on each of seven different zones, each classified based on the RayStation MLC model. The fractions, termed grid point fractions, were validated by analyzing simple test plans. The average grid point fractions, over all control points for 46 plans were then computed. These values were then compared with gamma analysis pass percentages and median dose differences to determine if any significant correlations existed.

**Results:**

Significant correlation was found between the grid point fraction metrics and median dose differences, but not with gamma analysis pass percentages. Correlations were positive or negative, suggesting differing model parameter value sensitivities, as well as potential insight into the treatment planning system dose model.

**Conclusions:**

By decomposing MLC control points into different transmission zones, it is possible to create a metric that predicts whether the analyzed plan will pass a quality assurance measurement from a dose calculation accuracy standpoint. The tool and metrics developed in this work have potential applications in comparing clinical beam models or identifying their weak points. Implementing the tool within a treatment planning system would also provide more potential plan optimization parameters.

## 1 INTRODUCTION

The calculation, transfer, and delivery of intensity modulated radiation therapy (IMRT) and volume modulated arc therapy (VMAT) plans on c‐arm treatment delivery systems are complex and have many potential points of failure. Hence, standard clinical practice has been to measure a test delivery of the same beams on a test object (phantom) and compare to a calculation. This process, while providing confidence that the treatment is as intended, is labor intensive and requires significant resources within the clinic. Recent research has also suggested that measurement‐based quality assurance (QA) practices may not be effective at detecting significant dosimetric errors.^1^ Because of this, a number of approaches have been explored to eliminate the measurement aspect and still maintain the same degree of confidence in the resulting treatment.

When the delivered dose does not meet intent, it can be due to inaccurate calculation, corruption of plan data, or the inability of the treatment delivery system to execute the delivery instructions. As an alternative to measurement, the analysis of either input (treatment plan) or output (log file) data has been considered. The first approach focuses on treatment plan multileaf collimator (MLC) positions. A number of plan complexity metrics based on MLC position data have been proposed to predict dose calculation accuracy relative to measurement.^2^ Several studies have also investigated the use of these complexity metrics as features for machine learning models of IMRT quality assurance result prediction.^3^, ^4^, ^5^, ^6^, ^7^, ^8^ The second analytic approach checks machine log files to see if the actual positions of machine components were at the intended set positions, again focusing on MLC position.^9^

Although a wide range of treatment plan MLC position analyses have been proposed, few have been able to definitively correlate with measurement results.^10^, ^11^ One aspect most share is the focus on leaf positions for each shape (control point) in a beam. The resulting metrics attempt to collapse a collection of one‐dimensional position information into a single value. In the process, a lot of differentiating detail can be lost, making it difficult to establish correlations between the metrics and measurement.

The accuracy of an IMRT/VMAT dose calculation depends heavily on the accuracy of the fluence model, and this depends on modeling the MLC transmission. In a treatment planning system, the different portions of the MLC are considered in defining the fluence model. For example, in the Eclipse (Varian Medical Systems, Palo Alto, California) and RayStation (RaySearch Laboratories, Stockholm, Sweden) treatment planning systems, a two‐dimensional (zero‐height) plane is divided into various zones representing the MLC leaf positions at each control point. In addition to the specific representation, the dose calculation depends also on one or more user‐adjusted parameter values optimized during commissioning. For example, the user may assign each zone a transmission value, and sometimes has control of zone dimensions.

We seek to create a tool that can analyze MLC transmission maps from plan delivery instructions generated by the treatment planning system (TPS). The tool constructs two‐dimensional arrays that are populated by analyzing plan data in the context of the TPS that created it, by decomposing a given plan's control points and identifying the contributions of each zone in the TPS MLC model to the fluence. Once we have the decomposition, we can compare the contributions of each zone to a QA measurement of that plan, and thereby attempt to establish a correlation. The tool not only records the relative contribution of each zone, but also the spatial extent in 2D, allowing for multi‐dimensional analysis within a single control point or across control points. In this study, we analyze the simplest case where a novel one‐dimensional metric is calculated from the two‐dimensional information the tool has collected. We propose that this analysis has predictive value and can be the basis for a potential tool to analyze treatment plan data a priori as part of an approach to eliminate the need to measure a given plan.

## 2 MATERIALS AND METHODS

We created a tool to analyze MLC positions in DICOM RT‐Plan files. While the idea behind the tool is TPS‐agnostic, we chose to make it compatible with the RayStation TPS for this work. It first classifies the two‐dimensional transmission map of a control point (segment) based on zones defined in the RayStation‐specific transmission array. Then it computes several metrics based on the relative area of each classified zone. Metric values were developed to establish a relationship with measured gamma analysis pass percentages and median dose differences for a suite of test VMAT plans created in RayStation.

### 2.1 Classification scheme

**FIGURE 1:**
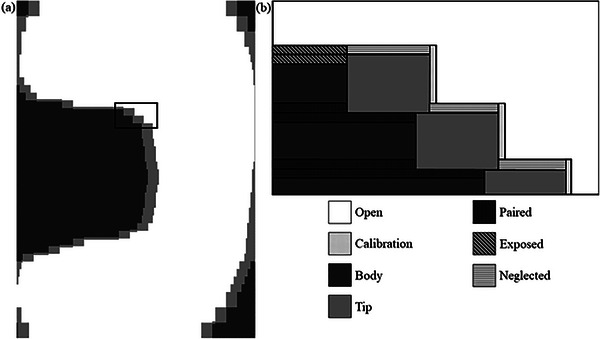
(a) Classification scheme applied to a control point from a clinical plan. (b) Close‐up providing zone detail.

The classification scheme is MLC‐ and beam model‐specific. In this work, it is based on the standard 120 leaf Millennium MLC on a TrueBeam (Varian Medical Systems, Palo Alto, California) c‐arm treatment delivery system and the corresponding institution's beam model within the RayStation 8B treatment planning system.^12^ In this model, the MLC is considered to have zero height and is represented as a 2D transmission array. It is sufficient to treat the MLC geometry of each control point as lying in a rectangular plane, the extent of which is defined by the jaw positions. The different zones for a representative single segment are illustrated in Figure 1. Zones within the plane are determined by which portion of the MLC leaves the beam passes through, corresponding to the open field, rounded tip, body, and tongue and groove portions of a physical leaf. Within RayStation, the tongue and groove zone is further divided into neglected, exposed, and paired sub‐zones, in order to account for different levels of transmission used in the model.

### 2.2 Decomposition tool

The tool was written in CPython 3.8.7 (www.python.org) and uses several modules from the standard library, as well as other modules such as matplotlib, numpy, and pydicom. The tool requires several inputs from the user. The primary one is a DICOM RT‐Plan file. The others are the RayStation MLC model parameter values, making the analysis specific to one RayStation beam model instance. We utilized a clinically optimized TrueBeam model for a flattened 6 MV (X06F) beam.^12^ The three coefficients of the calibration function (offset, gain, and curvature), tip width, tongue and groove width, and transmission parameter values are summarized in Table 1.

**TABLE 1:** RayStation model parameter value summary.

Parameter	Value
Offset (cm)	0.026
Gain	0.000
Curvature (cm^−1^)	0.000
Tip width (cm)	0.360
Transmission (%)	1.500
Tongue and groove width (cm)	0.040

#### 2.2.1 Scoring

With the DICOM RT‐Plan data and the RayStation parameter values, the scoring array for each control point was determined. The array is defined in the isocenter plane 100 cm from the source, perpendicular to the beam axis, with its boundaries being the projected jaw positions. Points in the plane were sampled on a grid using 0.1 mm spacing in both dimensions and assigned to one of the four general classifications: open, tip, body, or tongue and groove. First, preliminary tongue and groove zones of width twice the tongue and groove width parameter value were determined around the lines between neighboring leaves. Points belonging to the open or tip zones were then assigned. At this time, any points belonging to the preliminary tongue and groove group that did not belong to the open or tip classifications were confirmed to belong to the tongue and groove zone. Remaining points were then assigned to the body classification.

Once that was completed, points belonging to the tongue and groove zone were then assigned to one of the three subgroups: paired, exposed, or neglected. Similarly, specific points belonging to open field were reassigned to the calibration classification if their assignment changed after applying RayStation's position calibration.^12^ The assignment criteria for the seven classifications are provided in Table 2.

**TABLE 2:** MLC transmission zone classifications and their corresponding assignment criteria.

Classification	Criteria	Transmission
Open	Does not belong to leaf body, leaf tip, or tongue and groove zones of the MLC	1.000
Calibration	Belongs to open zone only after shifting the end of the leaves by distances calculated using the RayStation position calibration formula	1.000
Tip	Belongs to leaf tip zone defined by the post‐calibration leaf position, the leaf tip width, and the entire extent of the leaf in the direction perpendicular to the leaf's travel	√T
Body	Belongs to leaf body zone defined by end of the leaf tip, the jaw from which the MLC bank protrudes, and the perpendicular extent of the leaf minus the tongue and groove zones on both sides	T
Paired	Belongs to tongue and groove zone where the neighboring zones perpendicular to the leaf's travel are both not the open zone	T
Exposed	Belongs to tongue and groove zone where the neighboring zones perpendicular to the leaf's travel are body on one side and open zone on the other side.	√T
Neglected	Belongs to tongue and groove zone where the neighboring zones perpendicular to the leaf's travel are leaf tip on one side and open zone on the other side	1.000

The value for *T* is the RayStation model parameter value listed in Table 1.

For each control point, the fraction of the total number of points on the grid belonging to each classified zone were calculated. These grid point fractions (GPFs) were recorded, and this process was repeated for every control point of every beam of the plan starting with the first control point. Once all GPFs were acquired, various metrics were computed.

#### 2.2.2 Metrics

Using the collected GPFs, average values for each classification type were computed. The authors considered two ways to average the GPFs: the arithmetic average and a fluence‐weighted average, using the beam quantity (Monitor Units, MU) to represent the fluence. Additionally, these averages were calculated for each beam and the entire plan, allowing for analysis at the control point, beam, and plan levels. Weighting by the beam quantity delivered at each control point adjusts for the importance of each control point on the resulting dose distribution. Control points with a small number of MUs are less influential, while control points with more MUs may greatly affect the overall delivered dose. All average GPFs described in this work are the beam quantity‐weighted (fluence‐weighted) averages.

The average over the entire plan, most representative of the entire treatment, were calculated according to Equation 1:

(1) $$\mathrm{Average}\;\mathrm{GPF}\;=\frac{\sum_{i=1}^{N}f_{i}*m_{i}}{\sum_{i=1}^{N}m_{i}}.$$

where *f_i_* is the grid point fraction for the *i‐*th control point, *m_i_* is the beam quantity for the *i‐th* control point, and *N* is the total number of control points in the plan. This average was computed individually from the identified points in the 2D array for all seven classification types.

Additionally, the GPF for each control point was normalized by the open grid point fraction to help differentiate plans that have similar average grid point fractions, but significantly different open field areas. For example, this might occur when comparing a VMAT plan and a 3DCRT plan that happen to have similar jaw positions. The normalized averages are calculated according to Equation 2:

(2) $$\mathrm{Normalized}\;\mathrm{Average}\;\mathrm{GPF}\;=\frac{\sum_{i=1}^{N}\frac{f_{i}}{o_{i}}*m_{i}}{\sum_{i=1}^{N}m_{i}}.$$

The only difference in the two metrics is dividing by the open zone grid point fraction, *o_i_*, at each control point.

#### 2.2.3 Validation

**FIGURE 2:**
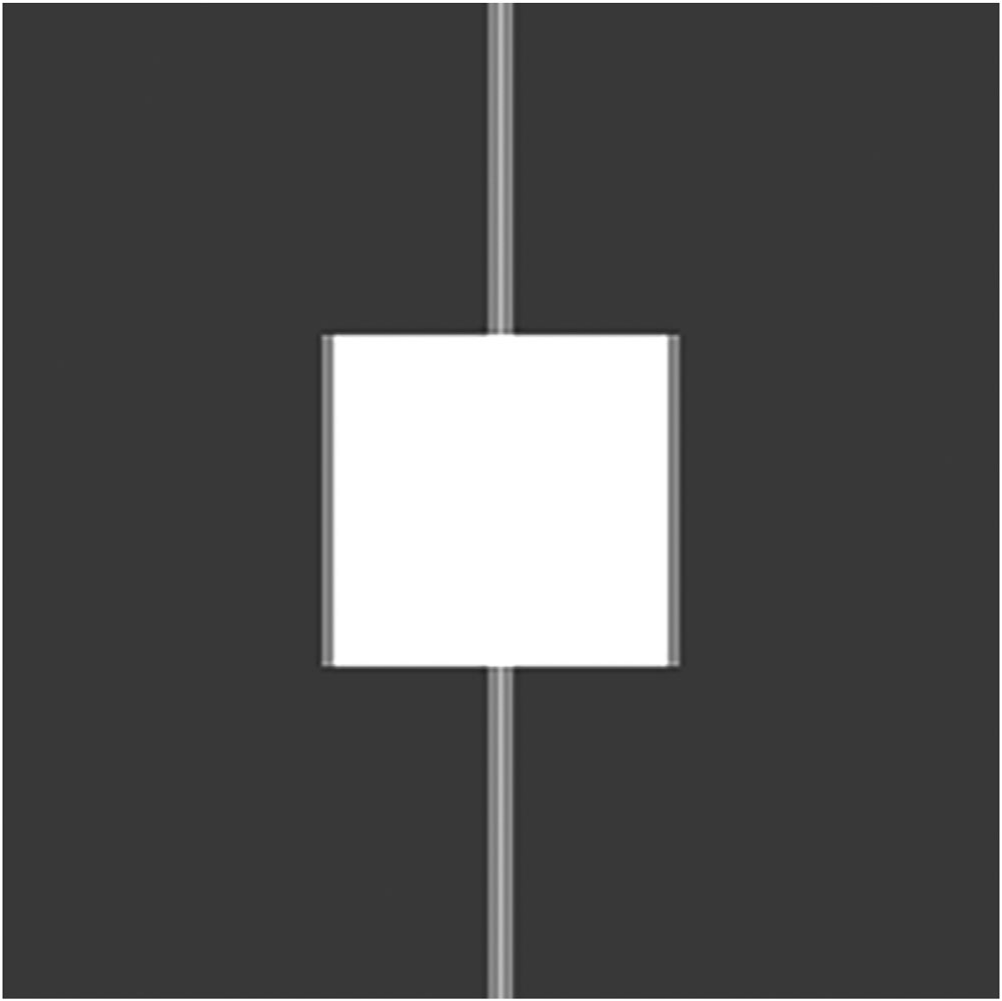
Test plan classification grid for one static beam. All leaves are positioned along the center of the field except for the 20 innermost leaf pairs, which are retracted to create a 10 × 10 cm^2^ zone of open field.

The GPFs and their corresponding averages were verified using a simple test plan which consisted of several beams. The first beam was a 30 × 30 cm^2^ square field, as defined by the jaws, with completely closed MLC leaves along the center of the field, except for one MLC leaf retracted by 0.5 cm, creating a 0.5 × 0.5 cm^2^ field. The remaining beams consisted of static square fields between 1 × 1 cm^2^ and 28 × 28 cm^2^. From these simple geometries, each grid point fraction could be calculated manually using known dimensions of the leaf tip, leaf body, and tongue and groove zones, and compared to the output of the Python tool. Figure 2 shows an image of the MLC arrangement for the fifth beam of the test plan.

### 2.3 Treatment plans

A total of 46 clinically treated plans were created in RayStation for a wide variety of treatment sites and techniques (e.g., lung SBRT, head and neck VMAT, and brain FSRT). Target volumes ranged from 2.8 to 2815.1 cm^3^. The diameter of a sphere of equivalent volume was calculated in order to provide an approximate reference of average target dimension.

### 2.4 Delivery quality assurance measurement

The three‐dimensional dose distribution for each plan was measured using a Delta4 Phantom+ system (Scandidos, Uppsala, Sweden).^13^ The gamma analysis passing percentage for a 2% local dose difference, 2 mm distance‐to‐agreement, 20% dose threshold was calculated. In addition, the median dose difference (MDD) for each plan for points above 50% of the maximum dose was also calculated, defined as

(3) $$\mathrm{MDD}=\frac{\left(Meas-Calc\right)}{Meas}.$$

All measurements were corrected for any machine output variation from the calibration entered in the TPS.

### 2.5 Correlation analysis

The gamma analysis pass percentage and median dose difference of each plan were plotted with respect to the plan average GPFs. Each scatter plot was fit using linear least squares regression. A *t*‐test for linear regression was performed for the various plots. The *t*‐test was executed in *R* (www.r-project.org). After inputting the data, the lm function was used to fit a linear model to the data. The summary function was called to determine details of the linear model. The summary provided the slope of the linear model, the standard error of the slope, its corresponding *t* value, and the probability that a test statistic exceeded |*t*|. This procedure was also performed for the normalized average GPFs.

## 3 RESULTS

### 3.1 Validation

The hand‐calculated GPFs were compared to the computed fractions. In all cases, the values were nearly identical, with all differences less than one part per million, significantly below the order of clinical significance. Any observed differences were the result of floating‐point precision errors.

### 3.2 Delivery quality assurance measurement

Plan properties and DQA measurement results are summarized in Table 3.

**TABLE 3:** Delivery quality assurance result and plan property summary.

Anatomical site	Target volume (cm^3^)	Sphere diameter (cm)	Gamma passing (percent)	Median dose difference (percent)
Esophagus	2.8	1.7	99.4	−0.9
Head	7.2	2.4	99.7	−1.1
Brain	13.1	2.9	99.8	−0.4
Rib	13.7	3.0	92.8	−1.7
Lung	15.4	3.1	99.7	−1.3
Brain	19.6	3.3	100.0	−1.6
Pelvis	21.9	3.5	79.6	−1.4
Brain	28.0	3.8	98.2	−1.6
Spine	50.9	4.6	98.5	−1.2
Prostate	51.7	4.6	99.7	−1.0
Spine	54.4	4.7	99.7	−0.7
Leg	57.9	4.8	97	−0.7
ChestWall	64.5	5.0	99.5	−0.6
Prostate	67.1	5.0	99.8	−1.0
Brain	72.9	5.2	94.2	−1.6
ChestWall	73.6	5.2	98.1	−0.9
ChestWall	74.9	5.2	99.5	−0.2
Mediastinum	80.7	5.4	82.8	−1.9
HeadNeck	120.9	6.1	96.1	−0.3
ProstateNodes	178.3	7.0	99.5	−0.2
Brain	185.9	7.1	97.7	−0.7
Prostate	188.6	7.1	100.0	−0.1
HeadNeck	204.0	7.3	84.1	0.6
ChestWall	221.2	7.5	89.2	−0.6
Esophagus	229.0	7.6	73.6	−1.3
Brain	247.7	7.8	94.2	−1.1
Breast	268.9	8.0	92.4	0.7
Brain	269.0	8.0	99	−0.5
Breast	301.5	8.3	73.2	1.3
Esophagus	315.5	8.4	96.3	−0.6
Brain	360.3	8.8	100.0	0.1
Pancreas	380.1	9.0	97.3	0.0
HeadNeck	429.0	9.4	95.4	0.2
Brain	443.0	9.5	99.8	0
Femur	471.9	9.7	98.5	−0.9
Lung	506.6	9.9	100.0	−0.2
HeadNeck	675.2	10.9	99.1	0
Leg	700.9	11.0	89.2	0.2
Breast	867.6	11.8	95.6	−0.2
Breast	867.6	11.8	95.2	0.5
Breast	1286.4	13.5	97.4	−0.4
Thorax	1297.7	13.5	95.5	0.2
Scapula	1952.2	15.5	96.6	−0.7
Scapula	1952.2	15.5	96.9	−0.5
Anorectal	2655.2	17.2	90.2	−0.3
Anorectal	2815.1	17.5	100.0	0.1

### 3.3 Average grid point fractions

**FIGURE 3:**
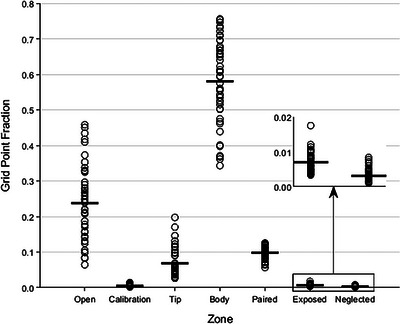
Average grid point fractions for all plans. The solid black lines indicate the mean of the values. The points for a given plan add to 1.0.

Figure 3 shows the average GPFs for all 46 plans. The open, tip, body, and paired grid point fractions show the largest range of values, indicating that the metrics may be useful in differentiating between the plans. However, the calibration, exposed, and neglected grid point fractions were all clustered in a small range of values. This was expected, as those zones are small in all plans.

### 3.4 Correlation analysis

#### 3.4.1 Gamma pass percentages

**FIGURE 4:**
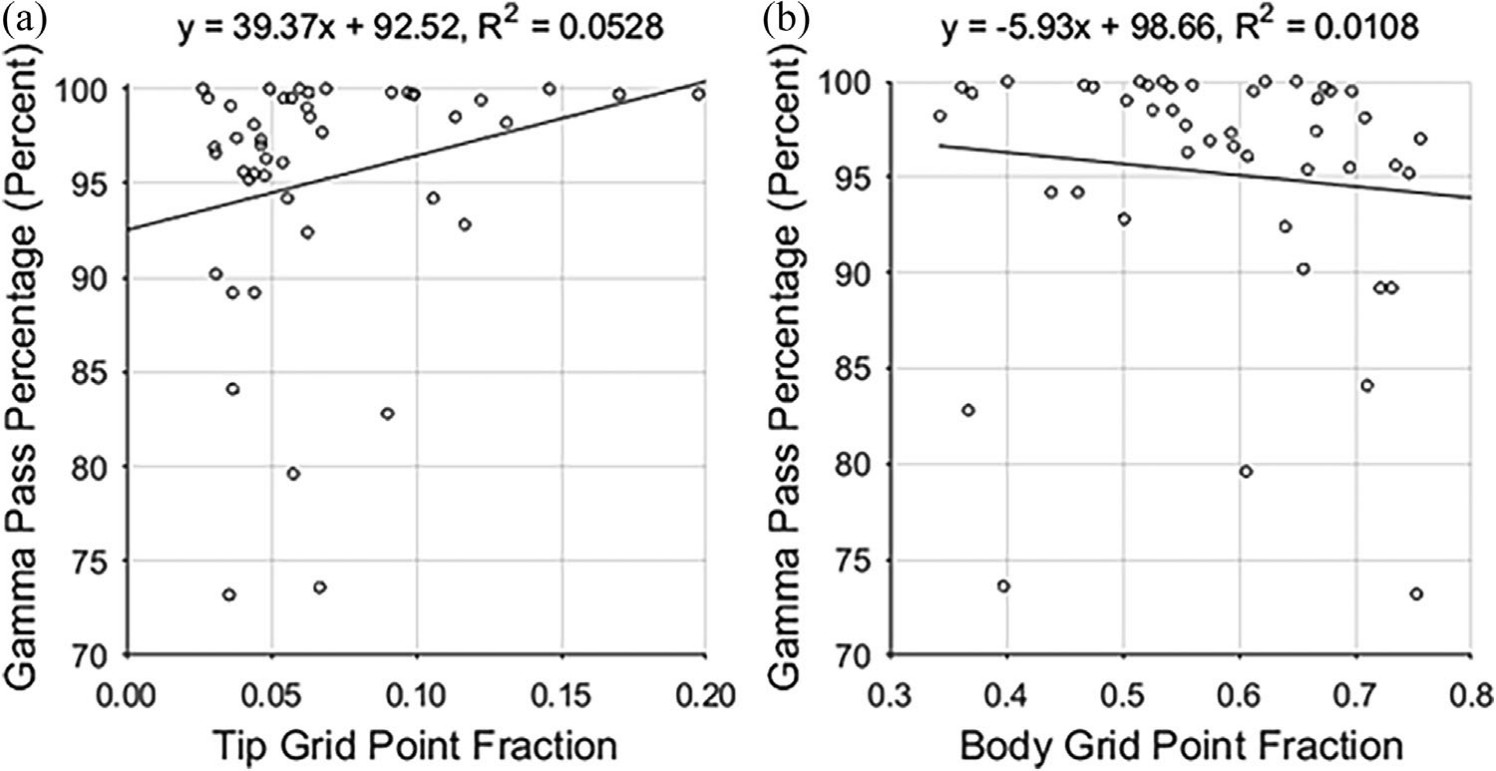
Gamma analysis pass percentages versus (a) average leaf tip grid point fractions and (b) average leaf body grid point fractions.

Figure 4 shows the gamma pass percentages versus the average tip and body GPFs. There is weak correlation between gamma pass percentages and the two metrics. A *t*‐test for linear regression was performed for each plot. Corresponding *p*‐values of 0.125 and 0.491 were obtained, indicating that the slope of the line of best fit is not statistically significantly different from zero. This means that we cannot reject a null hypothesis that there is no association between gamma analysis pass percentages and the average grid point fractions. Average GPFs for the classification types not shown were also not correlated with gamma analysis pass percentages.

#### 3.4.2 Median dose difference

**FIGURE 5:**
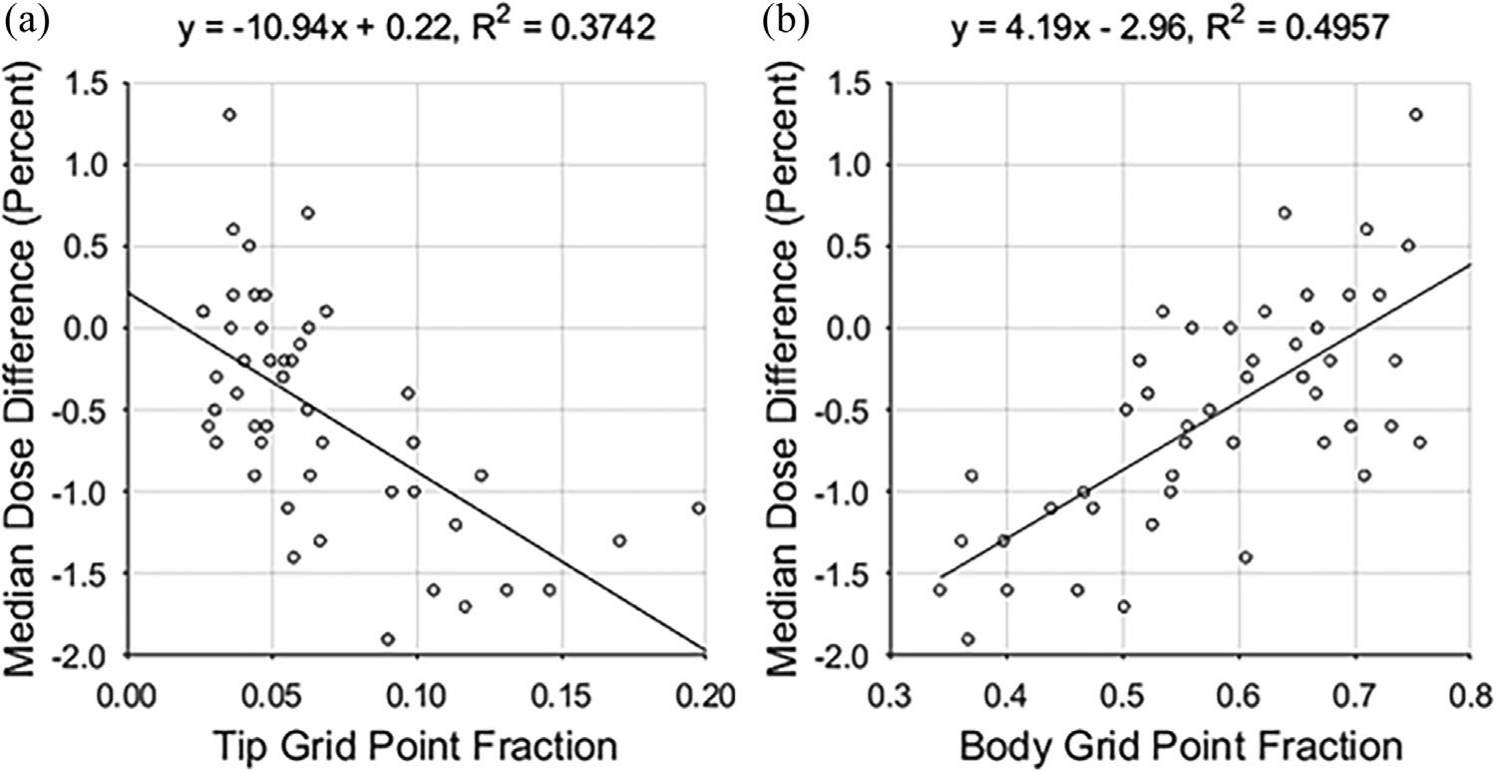
Median dose difference versus (a) average tip grid point fractions and (b) plan MU‐weighted average body grid point fractions.

**FIGURE 6:**
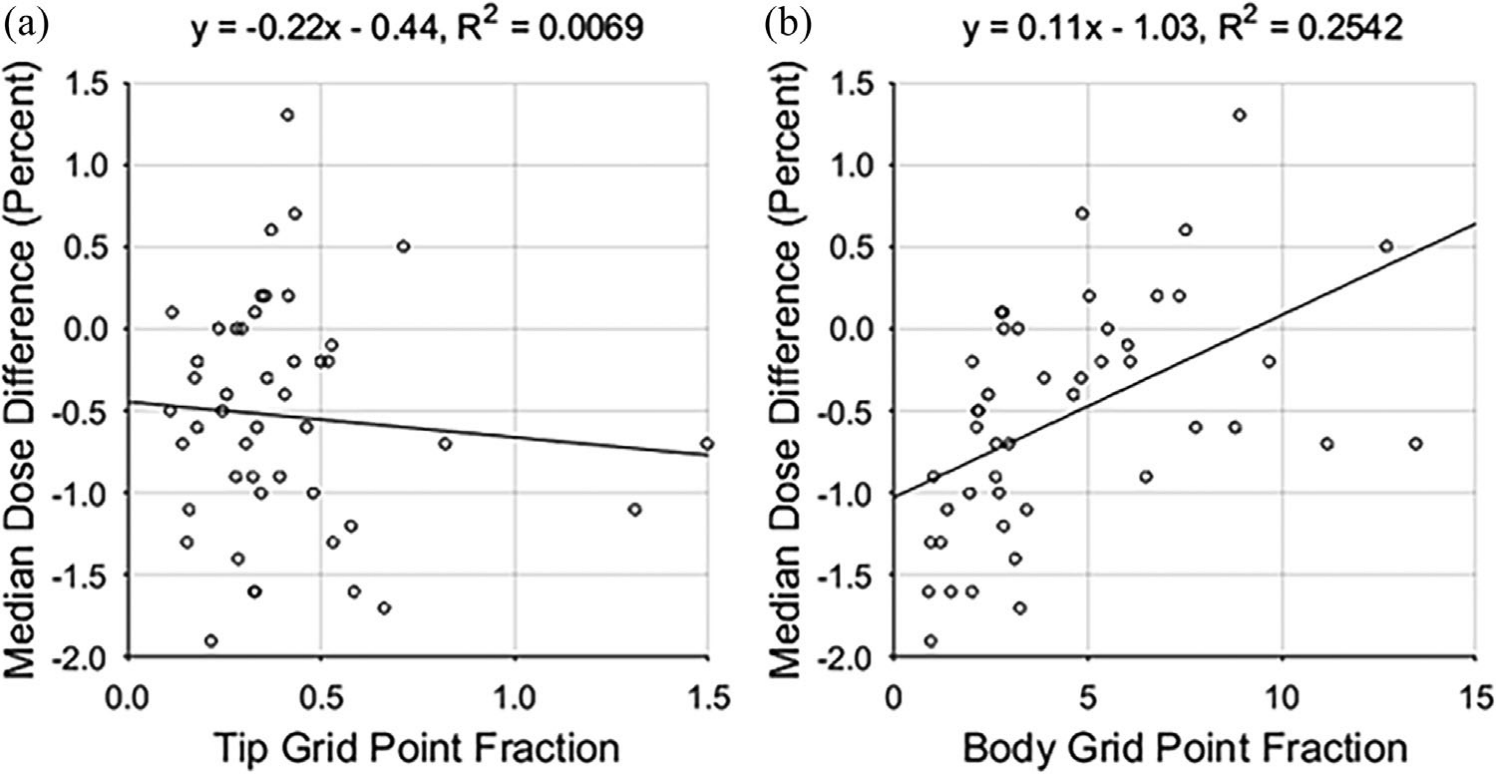
Median dose difference versus (a) plan MU‐weighted average tip grid point fractions and (b) plan MU‐weighted average body grid point fractions, both normalized by the open zone grid point fractions.

In contrast, median dose difference was found to correlate with two average GPFs, those of the tip and body zones. Plots of the relationships can be found in Figure 5. The correlation between average GPFs and median dose difference is stronger than with gamma analysis pass percentages. This is reflected in the *t*‐test for linear regression, with *p*‐values of 6.29 × 10^−6^ and 4.78 × 10^−8^. In both cases, there is significant evidence that there is an association between median dose differences and the average GPFs. This correlation is reduced after normalizing for the open zone grid point fraction, as shown in Figure 6.

## 4 DISCUSSION

### 4.1 Tool performance

From the analysis of the simple test beams, the tool produced GPFs correctly, as verified by hand calculations, and could be used with more complex beams. For the beam model parameter values used in our analysis, the average GPF values for several classifications, such as the body and tip zones, encompassed a wide range of values. This suggests that the GPF metric is useful in differentiating various treatment plans from each other. Plans that appeared significantly different at a glance, like those for different treatment sites or target volumes, but had similar average GPF values, were found to have similar QA results. The GPF metric values are smaller for zones that are of inherently small extent, such as the exposed and neglected zones. This is consistent with these zones not having many points relative to the others. In its current prototype state, the tool requires a couple of minutes to several hours to evaluate one plan. Increased efficiency through modifications to the code or faster hardware, for example, GPUs, would be necessary for clinical implementation if quick results are desired.

It should also be noted that the analysis in this study is focused on the GPFs and their relationship with QA measurement results. However, it is possible to use the two‐dimensional arrays of each zone for each control point to determine more complex relationships between VMAT plans and QA metrics. For example, one could analyze the frequency of overlap between points that belong to the tip zone and those with gamma greater than one. Texture analysis, for example, can be applied to the 2D maps for each zone type. This example and other ideas are avenues of future research related to the current project.

### 4.2 Correlation analysis

There was weak correlation between average GPF and gamma pass percentage values, as indicated by the R^2^ values in Table 4. This is not an unexpected result, as it has been observed for complexity metrics in the past.^14^ Gamma pass percentage is insensitive to small dose calculation inaccuracies resulting from the fluence model of the MLC. It is well known that the gamma metric is sensitive to noise and other fluctuations, which can bias the metric value downward and hence passing percentages upward.^15^ It is difficult to glean information from an insensitive metric that is the result of collapsing information. However, some groups have observed moderate to strong correlation between various complexity metrics and gamma pass percentages. Modulation index and plan averaged beam irregularity were found to strongly correlate with gamma pass percentages using a 2%/2 mm criterion in a study of 52 VMAT plans.^16^ Another study found moderate correlation (0.30 < *r* < 0.65) between various textural features and gamma pass percentages (2%/2 mm).^17^

**TABLE 4:** Correlations (*R* ^2^) of classification metrics with gamma pass percentages (GPPs) and median dose differences (MDDs).

	Metric	
Classification	GPP	MDD
Open	5 × 10^−5^	0.3722
Calibration	0.0521	0.3809
Calibration normalized	–	0.0103
Tip	0.0528	0.3742
Tip normalized	–	0.0069
Body	0.0108	0.4957
Body normalized	–	0.2542
Paired	0.0091	0.3108
Paired normalized	–	0.2037
Exposed	0.1352	0.2334
Exposed normalized	–	0.0143
Neglected	0.0778	0.3928
Neglected normalized	–	0.0449

In contrast, the median dose difference was found to strongly correlate with average GPF, with *r* > 0.50 for six of the seven zones. The GPF metrics outperform several metrics such as plan irregularity, plan modulation, and plan normalized MU, which have been found to have weak correlation with dose discrepancy.^18^ Other metrics like the converted aperture metric and the edge area metric have shown a stronger correlation (*r* around 0.9) with 5% dose difference pass rates than the GPF metrics with median dose differences show.^19^ In the future, the complexity metrics developed by other groups could be calculated for our data set of VMAT plans, and the corresponding correlations with gamma pass percentages and median dose differences could be evaluated and compared.

The correlations observed in this study suggest that the analyzed beam model produces calculation results which can be an over or underestimation of the delivered dose, depending on the contributions of the classification zones in the plan's beams. For the parameter values used in our analysis, most of the median dose differences are negative, meaning that the delivered dose was less than the calculated dose. Notice that for a large tip GPF, the dose differences are more negative than for a small tip GPF, while the opposite holds true for average body GPF. The model overestimates the dose delivered to the patient when the MLC tips compose a significant portion of the beam area. The relative transmission of the leaf tips is likely less than the single transmission value assigned to them in the TPS. By similar logic, the TPS likely models transmission through the body of the MLC leaves accurately, or if anything, the transmission is higher in reality than in simulation.

Normalizing the average GPFs by the open GPFs greatly reduced observed correlations with median dose differences. The purpose of normalizing the average GPFs was to discriminate between plans that had similar jaw positions. As the size of the arrays for each control point were determined by the space between the jaws, GPFs for some zones could be nearly identical between different plans. For example, if two plans had the same jaw positions, and all MLC leaf tips were entirely within the area defined by the jaws throughout the entire plan, then those two plans would have the same tip GPFs. However, the plans may have significantly different open zones, for example, if the target volumes of the two plans were unalike. It appears that only the relative area of the zones has an effect on the median dose differences and normalizing by the open GPFs confounds the correlation between MDD and average GPF.

Using the correlation analysis results, a clinical user could identify which regions of the MLC most affect the accuracy of their dose calculation. One could also compile a database of GPF values for plans at their institution. Plans with GPF values that fall within certain ranges would be more likely to have acceptable QA results. GPF results do not replace QA measurements, but they do predict which plans are more likely to need to be measured as part of the QA process.

The analysis performed in this study only involved one clinical beam model. It would be important for anyone hoping to apply this analysis at their own institution to determine correlation between the average GPF metrics and QA results using their own beam model instance. The performance of different beam models (parameter value sets) for a given TPS could be compared using analysis results.

### 4.3 Plan sensitivity

Prior studies have looked at the sensitivity of dose calculation to RayStation parameter values for a single plan.^20^ Conversely, the approach in this work provides an indicator of the sensitivity of a set of many plans to a single set of model parameter values. We can segregate plans by focusing on the variation of each zone GPF with median dose difference.

Specifically, one can look at the magnitude of the linear fit slope and its sign. As discussed earlier, the magnitude tells us how much the MDD will vary with GPF. A large slope value tells us that for a plan with a high zone GPF, the model parameter values for that zone significantly influence calculation agreement. When optimizing parameter values, the physicist should pay close attention to the model parameters that influence those zones. The sign of the slope indicates which direction the parameter value adjustments must go in the optimization process. As shown in Table 5, we find, in decreasing order, that neglected, calibration, exposed, and tip zones have the negative slopes. The paired and body slopes are smaller and positive. In general, as these slope values decrease, the areas of the zones increase. Small zones can have a big impact on the dose calculation.

**TABLE 5:** Slopes of lines of best fit for plots of classification metrics with GPPs and MDDs.

	Metric	
Classification	GPP	MDD
Open	−0.44	−4.05
Calibration	516.24	−145.67
Tip	39.37	−10.94
Body	−5.93	4.19
Paired	36.31	22.12
Exposed	818.89	−112.27
Neglected	996.40	−233.67

It would be valuable to perform this analysis for different beam models, that is, parameter value sets. Similarly, the dose calculation algorithm used may have an influence on the results. However, as the fluence map has a more substantial effect on the dose distribution than the dose calculation algorithm itself, we would expect that the results for a specific beam model would not be significantly altered. One could also extend the analysis beyond 6 MV flattened beams to different energies, unflattened beams, or different treatment machines. The analysis in this work could also be repeated for multiple regression with average GPFs for several classifications.

We conclude by noting that the classification scheme used in this work is specific to the RayStation TPS. However, the framework for use with other treatment planning systems exists within the tool, so this analysis could be repeated for plans generated using another TPS, for example, Eclipse. Additionally, it is conceivable that the tool could be implemented within a TPS, and the average GPF or other metrics based on GPFs or zone scoring could be used to formulate plan evaluation criteria. It also lends to creating beam model parameter value optimization objectives in the TPS physics module, whereby, for example, one could optimize against a plan spectrum until the slope of the GPF versus MDD line were flat and near a MDD of zero.

## 5 CONCLUSIONS

In this work a novel tool was introduced that can decompose individual control points from treatment plans into zones defined by what region of the MLC model those zones belong to. Using this tool, we have calculated a novel metric, the grid point fraction, and examined the correlation between the metric values for each zone and common QA measurements. Strong correlation was found to exist between average grid point fraction and median dose difference for the plans analyzed. The tool has predictive power in determining QA results, making it possible to anticipate which treatment plans are more likely to fail QA. For a given RayStation beam model, repeating the analysis performed in this work would help identify which zones of the MLC are more likely to lead to clinically significant dose calculation inaccuracies. The tool could have practical utility in the clinic when optimizing beam model parameter values. It is also conceivable that the tool be implemented within a TPS to use average GPF values as plan optimization parameters.

## AUTHOR CONTRIBUTIONS

Sean Frigo was responsible for the original idea behind the research. David Adam wrote a prototype of the decomposition tool. John Stasko refined the decomposition tool, collected all data, and performed the scientific analysis. Sean Frigo and Wesley Culberson oversaw the work. All authors met biweekly to discuss project updates and the future direction of the research. All authors were involved in writing and editing this manuscript.

## CONFLICT OF INTEREST STATEMENT

The authors declare no conflicts of interest.
